# Towards breaking the curse of dimensionality in computational methods for the conformational analysis of molecules

**DOI:** 10.1186/1471-2105-15-S3-A2

**Published:** 2014-02-11

**Authors:** Han Cheng Lie

**Affiliations:** 1 Institute of Mathematics – Freie Universität Berlin, Berlin, Germany

## Background

In computational molecular biology, the conformational analysis of a molecule refers to the identification of conformations - the clusters of molecular configurations sharing a large-scale geometric structure - and the description of conformation dynamics. These data help to describe the structures and functions of molecules which are relevant in biology. In applications such as computational drug design, they help to identify suitable compounds for disrupting processes involved in disease mechanisms. Today, many computational methods for conformational analysis are mesh-based: they involve discretizing configuration space using regular lattices, and simulating trajectories on energy landscapes. These methods suffer from the ‘curse of dimensionality’ in that the cost increases exponentially with the number of atoms in the molecule. Given that many important molecules in biology - such as proteins - have thousands of atoms, the curse of dimensionality severely limits the applicability of these methods. Finding an alternative approach to conformational analysis that does not suffer from the curse of dimensionality would enlarge the set of molecules and biological processes which may be studied using computational methods.

## Results

We construct a mesh-free method [1] using random sampling of energy landscapes, Monte Carlo quadrature, and coarse-graining [2]. We provide proof of concept by testing the method on a toy model of a molecule from the literature and find that the results of our method are comparable to those obtained by an exact, mesh-based method. Using results from linear programming theory and discrete geometry, we establish that the computational cost of our method increases only polynomially with the size of the molecule – an improvement over the exponential increase in the case of mesh-based methods.

## Conclusions

Our findings demonstrate that one does not need meshes for conformational analysis. For the specific problem we study in our paper – that of finding conformations and computing conformation transition rates – our findings suggest that mesh-free methods may be just as accurate as mesh-based methods, while costing less computationally. Our findings emphasize the utility of using ideas from different mathematical fields when devising new methods to tackle computational challenges.
